# Epigenetic Aberrations in Multiple Myeloma

**DOI:** 10.3390/cancers12102996

**Published:** 2020-10-15

**Authors:** Cinzia Caprio, Antonio Sacco, Viviana Giustini, Aldo M. Roccaro

**Affiliations:** CREA Laboratory, Clinical Research Development and Phase I Unit, ASST Spedali Civili di Brescia, 25123 Brescia, BS, Italy; capriocinzia@gmail.com (C.C.); saccoantonio74@gmail.com (A.S.); viviana.giustini@gmail.com (V.G.)

**Keywords:** multiple myeloma, epigenetics, DNA methylation, histone acetylation, non-coding RNAs

## Abstract

**Simple Summary:**

Multiple Myeloma (MM) is a blood cancer characterized by an uncontrolled growth of cells named plasma cells, within the bone marrow. Patients with MM may present with anemia, bone lesions and kidney impairment. Several studies have been performed in order to provide an explanation to how this tumor may develop. Among them, the so called “epigenetic modifications” certainly represent important players that have been shown to support MM development and disease progression. The present article aims to summarize the current knowledge in the specific are of “epigenetics” in MM.

**Abstract:**

Multiple myeloma (MM) is a plasma cell dyscrasia characterized by proliferation of clonal plasma cells within the bone marrow. Several advances in defining key processes responsible for MM pathogenesis and disease progression have been made; and dysregulation of epigenetics, including DNA methylation and histone modification, has emerged as a crucial regulator of MM pathogenesis. In the present review article, we will focus on the role of epigenetic modifications within the specific context of MM.

## 1. Introduction

Multiple myeloma (MM) is an incurable and biologically heterogeneous plasma cell dyscrasia which accounts for about 10% of all hematologic cancers [1]. It is characterized by an uncontrolled proliferation of plasma cells (PCs) within the bone marrow (BM), leading to extensive production of non-functional monoclonal immunoglobulin protein. The malignant plasma cells primarily colonize the BM, and patients with active MM present with a variety of clinical features that may include anemia, renal insufficiency, bone lesions, and hypercalcemia [2]. MM is consistently preceded by a pre-malignant condition named monoclonal gammopathy of undetermined significance (MGUS), occurring in about 3–5% of the population above 50 year of age [3]. Approximately 1% of patients/year may progress from MGUS to active MM [4]. MM cell may present with different genetic alterations [5,6]. Both chromosomal translocations and aneuploidy represent primary events associated with MM pathogenesis. The most frequently observed translocations involve the immunoglobulin heavy chain (IgH) locus: IgH gene is juxtaposed to oncogenes, thus resulting in their upregulation. t(11;14), found in about 20% of all patients with MM, leads to enhanced expression of CCND1, thus favoring MM cell cycle progression and cell proliferation [7]. Other frequently observed translocations include t(6;14) (~21%); t(4;14) (~14%); t(14;16) (<5%); which will result in up-regulation of cyclin D3, FGFR3 and NSD2/WHSC1, c-maf, respectively [8]. Importantly, somatic mutations have been recently described in MM, with KRAS (~25%), NRAS (~20%), FAM46C (~11%), DIS3 (~11%), and TP53 (~8%) representing the most frequently mutated genes [9,10].

Of note, epigenetic aberrations may also support MM pathogenesis. The term “Epigenetics” was defined in 1942, by Sir Waddington as “the branch of biology which studies the causal interactions between genes and their products which bring the phenotype into being” [11]. It refers to those mechanisms that controls gene expression via chromatin remodeling phenomenon that, in turn, can be stably inherited in the absence of DNA sequence changes [12]. The chromatin is organized in functional units composed of DNA and highly basic proteins found in eukaryotic cell nuclei, and its functional unit is represented by the nucleosomes, wrapped around a histone octamer.

The histone core octamer is composed of 4 sets of dimers, interacting with each other via the “histone fold”. Histone H3 and H4 interact through one fold, thus forming a heterodimer; similarly, H2A and H2B dimerize, as the result of their interaction through another histone fold [13]. Of note, nucleosomes do not only represent a tool necessary for DNA packaging, but they also are dynamic structures that mirror the gene-expression pattern of a given cell [14]. Nucleosome stability and dynamics may be modulated by chemical changes of the histones, thus affecting the energy landscape of histone–DNA interactions, leading to increase of the DNA accessibility. These chemical modification may appear as post-translational changes that can be added or enzymatically removed: the major and most studied modifications include methylation, acetylation, ubiquitylation, phosphorylation, and ADP-ribosylation [15,16]. Overall, hyperacetylated chromatin regions are transcriptionally active, in contrast with hypoacetylated that are transcriptionally inactive [13].

In the present review article, we will focus on the role of epigenetic modifications in supporting MM biology.

## 2. Epigenetics in Multiple Myeloma: DNA Methylation, Histone Acetylation, Epi-microRNAs

The process of lymphopoiesis is regulated by the expression of lineage specific gene sets. Indeed, progenitor cells present with several mechanisms of control of gene expression, resulting in precise and specific transcriptional patterns in a dynamic manner. This is controlled by the epigenetic machinery. Within the context of MM, main common epigenetic mechanisms have been described to play a role in MM pathogenesis, including DNA-methylation and histone acetylation. In addition, more recently, the literature has referred to “epi-microRNAs”, as those microRNAs that may target epigenetic regulators, including DNA methyltransferases and histone deacetylases [17,18].

### 2.1. DNA Methylation

The best-known epigenetic marker is DNA methylation. It plays a critical role in controlling nuclear architecture and gene expression. DNA methylation occurs within specific areas, named as CpG islands, enriched in dinucleotide CpGs and located at the 5′-end of the regulatory region of several genes. DNA methyltransferases DNMT3A and DNMT3B establish new DNA methylation patterns, while DNMT1A is responsible for maintaining methylation patterns upon replication.

Global DNA hypomethylation has been initially described in human cancer; other studies have then identified hypermethylated tumor-suppressor genes [19,20,21], followed by the description of miRNA inactivation by DNA methylation [22,23]. The literature has defined how CpG-island promoter hypermethylation may target genes that are involved in cancer pathogenesis, acting as crucial regulator of cell cycle progression, cell proliferation and survival, cell-to-cell interaction, programmed cell death [24,25]. Of note, DNA methylation could be influenced by histone structure modifications that are commonly disrupted in cancer cells [13,26,27,28].

Recent studies have reported on DNA methylation aberrations within the context of MM, showing several DNA methylation patterns with identification of hypermethylated loci in aggressive subtypes, compared to healthy donor [29]. Kaiser et al. reported on epigenetically-inhibited tumor suppressor genes, demonstrating their prognostic significance in MM [30]. For instance, GPX3, RSBP1, SPARC, and TGFBI were shown to be epigenetically inactivated in MM samples, and their methylation status has been associated with overall survival, demonstrating their prognostic relevance. Importantly, their inactivation was significantly higher as MM progressed to plasma cell leukemia [30].

DNA methyltransferase inhibitors (DNMTi) are currently used to revert aberrant DNA methylation patterns. Both cytidine analogs 5-azacytidine (azacytidine) and 5-aza-2′deoxycytidine (decitabine) have shown to exert an anti-myeloma activity, supported by enhanced DNA damage, cell cycle arrest, and induction of MM cell apoptosis [31,32]. Currently, clinical trials evaluating the safety of DNMTi in combination with lenalidomide or dexamethasone are ongoing within the MM setting [33] .

The mechanisms responsible for altered MM cell DNA methylation are not fully described. A higher expression of the DNA methyltransferase, DNMT1, has been reported in clonal MM cells as compared to their normal cellular counterpart. Several studies have investigated whether aberrations in DNA methylation could be described as disease stage-specific, thus changing during disease progression [34]. Indeed, methylation aberrations may occur as early events in myelomagenesis, being described in MGUS patients, involving mainly CpG islands [35]. CpG islands appear to play a critical role in recruiting Polycomb repressive complex 2-*PRC2* to gene promoters [36]. Enhancer of Zeste homolog 2-*EZH2* encodes part of PRC2 complex catalytic component; it regulates genome-wide chromatin structure and transcriptome profiling via histone H3-lysine-27 methylation, thus resulting in chromatin condensation and repression of transcription [37]. EZH2-overexpression is responsible for the suppression of cell cycle control genes and is associated to poor prognosis in several tumors, including prostate, colorectal, and breast cancer [38,39]. EZH2-overexpression has been recently described within the context of MM, demonstrating its negative prognostic value: specifically, authors have reported on increased EZH2 during disease progression, going from MGUS, to smoldering myeloma, to MM [40]. It is important to consider also the occurrence of mutations of epigenetic enzyme-related genes. Up to 53% of MM patients may present with mutated histone acetylation-, methylation-, DNA methylation-, and chromatin remodeling-related genes (Table 1). These mutations were also shown to be of prognostic relevance, as they correlated with overall survival in MM patients [40].

The chromatin reader *PHF19* modulates transcriptional chromatin activity [41], with a peculiar role within the context of B-cell-to-plasma cell differentiation [42,43]. *PHF19* acts recruiting the PRC2, binding to H3K36me3, leading to EZH2 activation [44,45]. This process ultimately enhances gene repression promoting tumor cell growth, as demonstrated in several tumor types [46]. Therefore, *PHF19* role in modulating MM biology has been a matter of investigation. Expression of *PHF19* was reported as significantly associated with MM disease progression, showing a predictive value greater than *NSD2* [47], an oncogene frequently over-expressed in MM plasma cells harboring the high-risk t(4;14) translocation; and reported to modulate by the oncogene *NSD2* [48]. These findings prompted research groups to dissect the potential functional relevance of *NSD2*-inhibition in MM.

The multiple myeloma SET domain (MMSET), also known as *NSD2* or *WHSC1*, was identified for the first time as a potential candidate gene for the Wolf–Hirschhorn syndrome (WHS) [49]. It acts as a histone-modifying enzyme, and its abnormal expression in MM is driven by the t(4;14).

*NSD2* interacts with both histone-H3 and -H4, thus leading to H3K36-dimethylation and H4K20-trimethylation; of note, it also favors the function of histone deacetylase-1, -2, and histone demethylase LSD1 [50,51,52]. Within the specific context of MM, the accumulation of H3K36me2 levels, causes transcriptional activation of oncogenes, thus leading to MM cell growth and disease progression [51,52].

Moreover, miRNA-126* has been identified [53] as an NSD2-regulated miRNA, as shown in MM cells harboring t(4;14). Of note, c-MYC is one of the miRNA-126*-predicted targets.

Studies have demonstrated how NSD2 binds to miRNA-126*-promoter, with the nuclear corepressor KAP-1 and HDACs; induces H3K9-trimethylation; reduces histone-H3 acetylation; thus, ultimately resulting in miRNA-126* silencing. Moreover, considering c-MYC as one of the miRNA-126*-predicted targets, the NSD2-dependent inhibition of miRNA-126* could result in increased c-MYC expression, thus contributing to MM pathogenesis [53].

Authors have demonstrated the anti-MM activity exerted upon NSD2-silencing using t(4;14)+ KMS11 cells, with a doxycycline-inducible NSD2-specific shRNA: in vivo studies confirmed reduction of tumor growth and enhanced survival in the setting of NSD2-silenced MM cells [54].

To further define the oncogenic relevance of *PHF19* in supporting MM biology, functional studies have been carried out and *PHF19*-silencing approaches have demonstrated inhibition of MM cell cycle progression, reduction of MM cell proliferation and survival [47], thus recapitulating the importance for *PHF19* in supporting MM pathogenesis and disease progression. Overall, targeting the *PHF19-PRC2-EZH2* complex could represent a novel therapeutic strategy for MM treatment. Indeed, EPZ-643 and GSK-126, known as EZH2 inhibitors, were shown to sensitize MM cells to HDAC inhibitor panobinostat, favoring MM cell apoptosis and reducing MM cell survival [55]. Similarly, recent studies have demonstrated how *PRC2* inhibitors were able to inhibit MM tumorigenicity [54,55]. It can be also hypothesized that *PHF19*-targeting could represent a novel therapeutic approach, considering its link to *PRC2* and *EZH2,* and given the anti-MM activity exerted by single and specific *PRC2-* or *EZH2*-targeting.

The expression levels of other histone methyltransferases (HMTs), such as members of the KMT1 and KMT2 families of lysine methyltransferases, have been reported to be altered in MM. For instance, the histone-lysine N-methyltransferase KMT1 member, SUV39H1, has been associated with tumor suppressor silencing in acute myeloid leukemia [56]. Similarly, its role in supporting MM pathogenesis has been also reported. SUV39H1 is differentially expressed between healthy donor- and MM-derived plasma cells; and high-SUV39H1 levels have been associated with adverse prognosis in MM patients. Importantly, the selective inhibition of SUV39H1, achieved using either small molecule or conditional shRNA-based approaches, is responsible for reduced MM cell proliferation, cell cycle arrest, enhanced DNA damage, and induction of the apoptotic phenotype in MM cells [57].

KMT2 methyltransferases act as a complex including histone acetyltransferase CBP/p300, members of the SWI/SNF chromatin-remodeling complex, and the demethylase KDM6A, to ultimately function as transcription activators [58]. Recent studies have reported on the occurrence of mutations within the KMT2 complex components, even though a clear functional role remains to be elucidated.

KDM6A and KDM6B, also known as UTX and JNJD3, respectively, guide demethylation of the repressive mark H3K27me2/3: they have been reported to be mutated in around 10% of MM cases [59,60], and the presence of the mutations correlates with adverse prognosis [60,61].

Among HMTs, protein arginine N-methyltransferases (PRMTs) catalyze the addition of methyl groups to arginine residues of histone tails.

PRMT4 and PRMT5, for instance, could play a role in supporting MM pathogenesis. PRMT4 (also known as CARM1) is overexpressed in several solid tumors, including breast, prostate cancer, and hepatocellular carcinoma [62,63,64]. PRMT4 catalyzes the addition of methyl groups to arginine 17 and 26 of histone H3, as well as to non-histone proteins. Overall, this would lead to transcriptional activation [65], cell cycle progression [66], DNA damage response [67], and cell differentiation [68]. The oncogenic role of PRMT4 has been described MM, and PRMT4-silecing using selective inhibitors, such as EZM2302 [69] or TP064 [70], was proven to exert anti-tumor activity in preclinical models of MM.

PRMT5 has been described to be up-regulated in MM patients, as compared to healthy individuals and, importantly, high levels of PRMT5 are associated with reduced progression free survival and overall survival [71]. Studies have properly shown the oncogenic role of PRMT5 within the specific context of MM. Of note, EPZ015666-dependent inhibition of PRMT5 exerted anti-MM activity, as documented both in vitro and in vivo [71].

### 2.2. Histone Acetylation

Histones may undergo post-translational modifications (PTMs), such as acetylation [72]. Acetylation of histones represents one of the most well-described PTMs, resulting the major player in remodeling chromatin structure and in modulating gene transcription [73]. Under physiological conditions, there is a well kept balance between histone acetyltransferases (HATs) and histone deacetylases (HDACs). HATs are enzymes that catalyze the addition of acetyl-groups to a specific lysine residue located within the histone N-terminal tail, with the aim to: neutralize lysine’s positive charge; favor an open chromatin structure; increase the accessibility of transcription factors to promoters; and, ultimately, to favor gene expression. Acetylated histones also function as binding sites for proteins with bromodomain which often positively regulate gene expression [74]. In contrast, HDACs are enzymes that act, catalyzing the removal of acetyl groups, resulting in transcriptional repression [75].

HATs comprise two classes of enzymes: type A and type B. Type A- and type B-HATs are localized within the nucleus or the cytoplasm, respectively. Type A-HATs are responsible for histone acetylation. Type B-HATs mainly acetylate newly translated histones, thus facilitating their assembly into nucleosomes [76]. Besides their main role in mediating histone acetylation, both types may be involved in transferring acetyl groups to other proteins, including many several oncogenes and tumor suppressors, such as MYC, P53, and PTEN, thus altering their protein functions [77]. Both solid tumor and hematologic malignancies, including MM, may present with coding mutations involving HATs [78].

CREB-binding protein (CBP)/p300, a type A-HAT, is essential in physiological events, such as cell proliferation, differentiation, and programmed cell death; it may also play a role during tumor transformation [79]. For instance, mutations in HAT CREBBP and gene copy loss of CREBBP and EP300 have been identified in MM patients [80] (Table 1).

**Table 1:** Mutation of genes encoding for epigenetic regulators.

CATEGORY	GENE NAME	EPIGENETIC ACTIVITY	MUTATED CASES (%)	MUTATION TYPE	REF.
**DNA methylation**	*DNMT3A*	DNA methylation	5	LOF	[59]
**Histone methylation**	*KMT2A*	Histone H3K4 methylase	1.7	LOF	[61]
	*KMT2B*	Histone H3K4 methylase	1.3	LOF	[61]
	*KMT2C*	Histone H3K4 methylase	1.5	LOF	[61]
	*SETD2*	Histone H3K36 methylase	1.3	LOF	[59]
	*NSD2*	Histone H3K36, K27 demethylase	0.4	LOF	[61]
	*NSD3*	Histone H3K36 demethylase	0.9	LOF	[61]
	*KDM6A*	Histone H3K27 demethylase	1.3	LOF	[60,61]
**Histone acetylation**	*P300*	Histone H3K27 acetylase	1.3	LOF	[79,80]
	*CREBBP*	Histone H3K27 acetylase	0.7	LOF	[79,80]

LOF: loss of function.

Given the oncogenic role of the CBP/p300 HAT, several attempts have been made to develop novel therapeutic intervention; and the small molecule CCS1477 has been designed to selectively target and inhibit CBP/p300. Its anti-MM effect has been demonstrated both in vitro and in vivo, used either as a single agent or in combination with lenalidomide [81]. CCS1477 is currently approved for Phase I/II clinical trial for the treatment of MM.

Enhancer rewiring is a fundamental epigenetic mechanism in carcinogenesis and acetylation of H3K27 is critical in regulation of enhancers. For this reason, targeting enzyme binding acetylated histones has often been highlighted as a therapeutic strategy in MM. The bromodomain and extra-terminal domain (BET) superfamily consist of four proteins (BRDT, BRD2, BRD3, and BRD4), each containing two bromodomains, capable of recognizing acetylated lysine residues. BRD-proteins promotes the recruitment of transcriptional activators, thus enhancing transcriptional activation [82]. Abnormal transcription activation resulting from aberrant expression of transcription factor represents a crucial step for MM pathogenesis: for instance, MYC upregulation is seen in up to 50% of MM patients [83]. Importantly, selective BRD4-inbhition, via JQ1, has been proven to exert an anti-tumor effect in MM, as demonstrated in preclinical model both in vitro and in vivo [84]. The JQ1-dependent anti-MM activity was supported by induction of MM cell cycle arrest and cellular senescence [84]. The other class of enzymes that balance the activity of HATs are HDACs. Four HDAC classes have been identified (I, IIa, IIb, and IV), for a total of 11 canonical subtypes. Specific biological functions of each HDAC isoform result from a differential HDAC localization within the cell; tissue-specificity; enzymatic activity; and substrate specificities. Several biological processes are regulated by HDACs, including for instance, modulation of senescence, cell differentiation, cell survival, programmed cell death, angiogenesis [85] HDACs may present with aberrant expression and function, leading to enhanced cell proliferation and survival, as demonstrated within the context of tumor transformation [86,87,88,89,90]. Within the field of blood cancers, increased HDAC expression was reported in lymphoproliferative disorders (diffuse large B-cell lymphomas; T-cell lymphomas; cutaneous T-cell lymphomas; acute lymphoblastic leukemia) and myeloproliferative neoplasms [91,92,93,94,95]. Of note, the aberrant expression and function of HDACs has also been described in MM where Class I HDAC over-expression (i.e., HDAC1), was shown to be of prognostic relevance and associated to poor prognosis [93]. Overall, these studies provided the rational for considering HDACs as potential new targets for therapy. Indeed, several efforts were made to design and develop HDAC-inhibitors, in terms of both HDAC-specific blockade and pan-HDAC-inhibitors [96].

Histone deacetylase inhibitors (HDAC-Is), including for instance, Vorinostat and Panobinostat represent HDAC inhibitors that would therefore elicit a potential anti-tumor effect acting as epigenetic-targeting agents; and their activity has been also evaluated within the context of MM [97]. Extensive research has unveiled the ability of HDAC6-inhibition to halt protein degradation, leading to significant accumulation of polyubiquitinated proteins by targeting the aggresomal protein degradation machinery; thus ultimately favoring MM cell apoptosis. Of note, studies have also shown a synergistic activity between HDAC6- and proteasome-inhibition [98].

Vorinostat acts as a reversible inhibitor of class I and II HDACs [99]; and its anti-MM effects were demonstrated in MM models, resulting from Vorinostat-dependent inhibition of MM cell proliferation, enhanced MM cell apoptosis, reduced expression of pro-survival factors [100].

Panobinostat is a cinnamic hydroxamic acid analog that has proven to act with 10-fold higher inhibitory activity against Class I, II, and IV HDACs as compared to Vorinostat: it has shown anti-MM activity, either used as a single agent or in combination with proteasome inhibitors [101].

HDAC deregulation may be responsible for drug resistance, and indeed, studies have reported on HDAC1 over-expression as a possible player in mediating MM cell resistance to bortezomib-dependent proteasome inhibition [93].

Romidepsin acts as primarily class I HDAC inhibitory, and its anti-tumor activity has been described within the field of MM, showing synergism when used in combination with bortezomib [102,103]. More recently, authors have described the anti-MM activity exerted by ACY-241 (HDAC6 selective inhibitor), when used in combination with both immunomodulatory drugs (IMiDS) and proteasome inhibitors (PIs). In addition, importantly, ACY-241-dependent HDAC6 inhibition was supported by enhanced immune response in MM, resulting in a more efficacious host anti-tumor immunity when used in combination with PIs and IMiDS [104].

### 2.3. Epi-microRNAs and lncRNAs

Thousands of genes that lead to non-coding RNA (ncRNA) transcripts have been described in the past years in tumor cells, thus suggesting the presence of high complexity within the genome of cancer cells. Based on their length, they have been classified as short non-coding RNAs (<200 nucleotides, sncRNAs) or long non-coding RNAs (>200 nucleotides, lncRNAs) [105]. miRNAs are small non-coding RNAs (∼22 nt) that play crucial role under physiological and pathological conditions: they target mRNAs of protein-coding genes, thus negatively regulating their expression [106]. The biogenesis of miRNA involves a complex protein system. The RNA polymerase II (RNA pol II) transcribes the miRNA sequence present within the DNA, resulting in precursor miRNAs, named primary miRNAs (pri-miRNAs). At the nuclear level, the complex of Drosha (RNase III) and Pasha (DGCR8) convert pri-miRNAs into pre-miRNAs. Pre-miRNAs are subsequently exported from the nucleus to the cytoplasm, by exportin-5; and Dicer (RNA III Family member) acts to cleave the hairpin in dsRNA (including the mature miRNA guide, and the complementary passenger strand). The mature miRNA strand of the duplex is then assembled to form the ribonucleoprotein complex (RISC). The remaining strand typically undergoes degradation. The mature miRNA-containing RISC is able to target the related mRNAs by suppressing their translation [107,108,109].

The field of B-cell lymphoproliferative disorders has been the first area to be investigated in terms of miRNAs in cancer. Dr. Croce and colleagues were the first to identify tumor suppressors miRNAs, located at 13q14, where a significant lower expression of miRNA-15a and -16-1 was demonstrated in patients with chronic lymphocytic leukemia, as compared to their normal cellular counterpart [110]. Similar findings were also described within the field of MM, where lower expression of miR-15a and -16-1 was demonstrate in MM patients’ derived plasma cells [111,112]. These studies, reported, for the first time on the tumor-suppressor role of miR-15a and -16-1, in MM, demonstrating how miR-15a and -16-1 gain of function approached were able to inhibit MM tumor growth both in vitro and in vivo, using xenograft disseminated MM models [111,112].

Epigenetic modifications may be responsible for impaired miRNA expression. For instance, both DNA methylation and histone modifications have reported to modulate miRNA levels [113].

Overall, the functional relevance of miRNAs in supporting the biology of cancers is due to the presence of miRNAs within genome regions that encode for either oncogenes (OGs) or tumor suppressor genes (TSGs). In the first case tumor cells will present with reduced expression of the given miRNAs, thus leading to lack of miRNA-mediated OG silencing; while, in the second case, tumor cells will presenting with over-expression of a certain miRNAs, that will ultimately result in enhanced inhibition of the target TSG [114]. It is important to take into consideration how epigenetics, such as DNA methylation, may modulate miRNA expression [115]. Specifically, authors have demonstrated the presence of DNA methylated peaks within intragenic and intronic regions, in MM; and found hypermethylation-mediated inhibition of tumor suppressor-miRNA-10b-5p, and -miRNA-152, thus leading to overexpression of their target genes (oncogenes DNMT1, BTRC, MYCBP, and E2F3) in CD138+ bone marrow derived MM cells [29].

Epigenetic regulation of the tumor suppressor miRNA-23b has been reported in MM, resulting from the occurrence of methylation of its promoter region [116]. Specifically, the authors have reported on the miRNA-23b/Sp1/Myc feed-forward loop as a crucial regulator of MM cell growth; and proven how promoter methylation may represent one of the mechanisms supporting the suppression of miRNA-23b. Functional studies were also carried out, demonstrating how miRNA-23b-gain of function led to inhibition of MM cell proliferation and survival, supported by induction of caspase-dependent apoptosis [116]. miRNA-23b targets Sp1 3′UTR, thus resulting in inhibited Sp1-driven NF-kB activation. Moreover, the authors provide novel insights into the ability of the oncogenic transcription factor c-Myc to repress miRNA-23b. Therefore, c-Myc-induced miRNA-23b silencing may favor the oncogenic Sp1-activity, thus enhancing MM cell growth and survival.

Recent studies have focused on the role of miRNAs in regulating the epigenetic machinery. For instance, miRNA-29b has been shown to target DNMT-3A and -3B mRNAs, thus leading to global hypomethylation in MM cells [17]. miRNA-29b mimic-transfected MM cells presented with inhibition of cell cycle progression and reduction of MM cell growth. Importantly, in vivo studies have confirmed how systemic delivery of synthetic miR-29b mimics exerts significant anti-MM activity [17]. Importantly, miRNA-29b has been shown to target HDAC4 in MM cells, thus further confirming the role of miRNA-29b as an “epi-miRNA” in MM [18]. miRNA-29b-dependent inhibition of HDAC4 resulted in reduced MM tumor growth as demonstrated both in vitro and in vivo [18].

The role of lncRNAs in supporting MM pathogenesis has been recently reported. Among lncRNAs that could be involved in epigenetic regulation of MM disease, MALAT1, RP11-553 L6.5, ZFY-AS1, RP4-803, RP1-43E13.2, and BM742401 have been described. [18,117,118,119].

Higher expression levels of MALAT1 were associated with MM disease progression [120]. Of note, MALAT1-silencing strategies led to inhibition of MM tumor growth, thus providing the preclinical rational for considering MALAT-1 a novel therapeutic target in MM [117]. MALAT1 is able to induce up-regulation of epi-miRNAs, such as miRNA-29b [18], with an inverse correlation with EZH2 enhancer [118]. EZH2-inhibition results in enhanced expression of the epi-miRNA miRNA-29b, as a consequence of H3K27me3 in promotor regions of miR-29b [118].

RP11-553 L6.5, ZFY-AS1, RP4-803, and RP1-43E13.2 were also described as modulators of MM disease progression, as a result of their correlation with epigenetic changes connected to MM [119].

Another lncRNA that has been described as epigenetically silenced by DNA promoter methylation in MM is BM742401 [121]. Higher level of BM742401 methylation characterized MM cells, as compared to normal plasma cells, leading to reduced expression of BM742401. The tumor suppressor role of BM742401 was also demonstrated by performing BM742401 gain-of-function studies, unveiling inhibited MM cell migration, thus suggesting how epigenetic silencing of BM742401 may enhance myeloma metastases and disease progression [121].

Most recently, several studies have also focused on the role of novel assays for the detection of ncRNAs within several body fluids, thus suggesting the use of minimally invasive procedures for identifying miRNA patterns in MM [122,123,124]. For instance, circulating exosomal miRNAs were described as an important tool to enhance the stratification of MM patients with high-risk disease; and miRNA-let7b and -18a were associated with both progression-free and overall-survival [123].

In summary, miRNAs have certainly gained scientists’ attention due to their important within the clinical setting. Indeed, miRNAs may be used to differentiate between a tumor and a normal tissue; between tumor subtypes, within a given tumor type; to better define outcome of patients and their response to treatments; and, finally, can be either silenced or over-expressed for therapeutic purposes.

## 3. Role of Epigenetics in Supporting the MGUS-to-MM Transition

Epigenetic dysregulation represents a well-known hallmark of tumor cells, contributing to cancer onset and tumor progression. Detailed mechanisms underlying the MGUS-to-MM progression have not fully defined. Authors have defined a gene pattern for high-risk MM [125,126], nevertheless, additional studies are required for properly define mechanisms responsible for the MGUS-to-MM transition. Recent studies have dissected the potential role of epigenetics in supporting the MGUS-to-MM transition, including, for instance, DNA and histone methylation [34]. MM cells and several other tumor types present with global hypomethylation of DNA, together with hypermethylation of gene-specific promoter regions [127]. Genome-wide methylation arrays have been performed at several stages of MM disease, demonstrating the occurrence of DNA hypomethylation at early MM phases; importantly, the hypomethylated status was shown to further decline during MM progression [34]. Notably, promoter hypermethylation of several cancer-related genes (i.e., BNIP-3, p16, E-CAD, DAPK-1), was reported to correlated with adverse prognosis [30,128,129,130]. Recent studies have reported on the promoter methylation-dependent silencing of RASSF4 (RAS association domain family member 4), occurring with MM disease progression; with a correlation with adverse prognosis [131].

Transcriptome profiling has demonstrated EZH2 up-regulation during MM progression, with a specific enrichment within the high-risk proliferative subgroup [40]. Several evidences have highlighted the role of miRNAs in MM progression. Moreover, serum-miRNA signature was evaluated in both MGUS and MM patients, demonstrating its prognostic and diagnostic relevance [132,133].

Recent studies have dissected the potential role of methylation on miRNA expression; and reported on hypermethylation-mediated silencing of miRNAs that have reported as potential TS-miRNA in MM (i.e., miRNA-10b-5p, -152) [29].

Moreover, components of miRNA processing machinery are also associated with MM pathogenesis. Aberrant expression of Dicer has also been shown to play a role in the MGUS-to-MM progression; and to correlate with outcome in MM. Specifically, reduced Dicer levels were associated with disease progression from MGUS to active MMM; whereas increased Dicer levels resulted in prolonged PFS in MM patients [116,134]. In addition, the miRNA processing- and B-cell differentiation-regulator and Argonaut 2 (AGO2) [21,135,136] was shown to be up-regulated in MM patients with high-risk disease [126]. This findings prompted the authors to investigate the functional role of AGO2 in MM, demonstrating its oncogenic role, as shown by enhanced MM cell programmed cell death in AGO2-silenced MM cells [137].

## 4. Targeting Epigenetics within the Context of the Bone Marrow Milieu

High dose chemotherapy followed by stem cell transplantation, and the use of novel small molecules and antibodies, have certainly improved MM patient survival. Nevertheless, MM remains an incurable disease, and most patients succumb due to disease relapses and progression. It has been shown that the bone marrow (BM) milieu enhances MM cell survival and proliferation, conferring drug resistance [138]. How bone marrow mesenchymal stromal cells (BMSCs) may support MM disease progression and drug resistance has gained the attention of several research groups. For instance, BMSCs could favor the acquisition of additional genetic aberrations within the tumor clone, thus leading to prolonged MM cell survival, and drug-resistance, even in those cases presenting with a clinical remission state.

It has been previously reported that selective HDAC3-targeting using BG45, results in inhibition of MM cell growth [139]. Importantly, other groups have demonstrated how targeting HDAC3 within the context of the BM milieu results in an indirect anti-MM activity [140]. These data were supported the demonstration that HDAC3 levels were significantly higher in MM patients as compared to healthy individuals, and, importantly, MM cells were shown to induce the expression of HDAC3 in BMSCs [140].

Moreover, additional studies have also demonstrated the importance of engineering miRNAs in MM cells, aiming to obtain an anti-MM effect: indeed, enhancing the expression of pre-miRNA-15a and -16-1 in MM cells has shown to result in a significant anti-MM activity, supported by inhibition of MM cell adhesion to the surrounding BM milieu, resulting in disruption of the MM cell-to-BMSC cross talk, followed by significant reduction of MM tumor growth, as shown using xenograft disseminated MM models [112].

## 5. Conclusions

It is well accepted the concept that epigenetic alterations are an important process in MM pathogenesis and progression. All these findings yield potential avenues for novel therapy, thus suggesting the use of epigenetic-targeting drugs as a putative novel therapeutic approach for this disease. Several studies have dissected the potential anti-MM activity exerted by epigenetic modifier-targeting agents, such as inhibitors of DNMTs, HDACs, EZH2; and confirmed their anti-neoplastic effect in pre-clinical models of MM, as shown both in vitro an in vivo. Sequencing of MM patients during their disease progression could result in the identification of new epigenetic-related targets, thus supporting the rational for the design and development of new epigenetic-targeting agents for personalized therapies. Moreover, considering the recent advances within the field of CRISPR-Cas9-based genome editing approaches, these technologies could be potentially applied to better define the functional role of the described epigenetic aberrations, thus further contributing to a better understanding of the MM biology; and allowing for the identification of novel therapeutic interventions.

Furthermore, data from utilizing epigenetic inhibitors targeting several epigenetic modifiers e.g., DNMTs, HDACs, EZH2, BMI-1, and BET bromodomains have shown pleiotropic anti-MM effects by affecting several oncogenic pathways using MM in vitro and in vivo models. The use of relevant pre-clinical animal models, such as the Vk*MYC mice [141], that highly resemble the MM disease as it appears in human will be an absolute requirement to evaluate the efficacy and safety of epigenetic inhibitors in a syngeneic tumor microenvironment, as well as on-target drug effects in vivo. Such studies will provide proof-of-concepts for the translation of rational targeting of epigenetic modifiers into clinical practice in MM.
